# Drug resistance in targeted cancer therapies with RAF inhibitors

**DOI:** 10.20517/cdr.2021.36

**Published:** 2021-06-17

**Authors:** Ufuk Degirmenci, Jiajun Yap, Yuen Rong M. Sim, Shiru Qin, Jiancheng Hu

**Affiliations:** ^1^The Laboratory of Cancer Signaling, National Cancer Centre Singapore, Singapore 169610, Singapore.; ^2^The Cancer and Stem Cell Program, Duke-NUS Medical School, Singapore 169857, Singapore.; ^#^Authors contributed equally.

**Keywords:** RAS/RAF/MEK/ERK signaling, RAF/KSR family kinase, oncogenic mutation, targeted therapy, RAF inhibitors, drug resistance, regulatory spine

## Abstract

Hyperactive RAS/RAF/MEK/ERK signaling has a well-defined role in cancer biology. Targeting this pathway results in complete or partial regression of most cancers. In recent years, cancer genomic studies have revealed that genetic alterations that aberrantly activate the RAS/RAF/MEK/ERK signaling mainly occur on RAF or upstream, which motivated the extensive development of RAF inhibitors for cancer therapy. Currently, the first-generation RAF inhibitors have been approved for treating late-stage cancers with BRAF(V600E) mutations. Although these inhibitors have achieved promising outcomes in clinical treatments, their efficacy is abolished by quick-rising drug resistance. Moreover, cancers with hyperactive RAS exhibit intrinsic resistance to these drugs. To resolve these problems, the second-generation RAF inhibitors have been designed and are undergoing clinical evaluations. Here, we summarize the recent findings from mechanistic studies on RAF inhibitor resistance and discuss the critical issues in the development of next-generation RAF inhibitors with better therapeutic index, which may provide insights for improving targeted cancer therapy with RAF inhibitors.

## INTRODUCTION

RAS/RAF/MEK/ERK signaling plays a determinant role in cell fate and is tightly regulated in normal cells^[1-3]^. Hyperactivation of RAS/RAF/MEK/ERK signaling by germline mutations induces developmental disorders termed as RASopathies^[4,5]^, whereas that by somatic alterations causes human cancers^[6,7]^. In human cancers, the majority of genetic alterations that aberrantly activate this pathway occur on receptor tyrosine kinases (RTKs), NF1, RAS, and RAF^[8-10]^. Oncogenic RTK alterations can be effectively targeted by tyrosine kinase inhibitors (TKIs) or neutralizing antibodies^[11-14]^, while active RAS mutations except ~10% G12C have been thought as “undruggable” targets^[15,16]^. As the first kinase of this signaling cascade, RAF is thus an ideal target for developing drugs against cancers with RAS or RAF mutations as well as loss-of-function of NF1 tumor suppressor^[17-20]^. Indeed, the first-generation RAF inhibitors have been developed and applied to clinical cancer therapy^[21-23]^. Although these inhibitors have no effect on cancers with active RAS, they have achieved promising outcomes on cancers with BRAF(V600E), a dominant mutation of RAF in cancer genomes. However, their efficacy is completely abrogated by acquired resistance in 6-7 months^[24-27]^. Mechanistic studies have suggested that both intrinsic and acquired drug resistance arises largely from the unexpected homo- or hetero-dimerization of RAF proteins^[28-33]^. These findings indicate that the regulation of RAS/RAF/MEK/ERK signaling is much more complicated than anticipated and its understanding will help us develop novel RAF inhibitors with long-term efficacy, broad coverage, and minor side-effects. In this review, we discuss key issues in targeted cancer therapy with RAF inhibitors and the development of next-generation RAF inhibitors, which may facilitate targeted therapy against cancers with RAS/RAF mutations.

## THE RAS/RAF/MEK/ERK SIGNALING IN CELL BIOLOGY

The RAS/RAF/MEK/ERK pathway is fundamental to cellular proliferation, differentiation, and survival in response to growth signals from extracellular environment^[3,34]^. The discovery of this pathway dates back to the 1970s when studies of viral oncogenes from sarcoma viruses identified RAS small GTPases^[35-38]^. Shortly after the discovery of RAS, an N-terminal truncated version of CRAF (RAF1) was isolated from murine and avian retroviruses^[39,40]^. In 1984, it was uncovered that epidermal growth factor (EGF) activated RAS and that RAF functioned downstream of RAS^[41-43]^. Subsequently, the EGFR-RAS-RAF link was further confirmed by studies done in *Drosophila* and *C. elegans*^[44,45]^. Downstream of RAF, MEK and ERK were identified as cytoplasmic protein kinases activated by mitogens in the 1990s^[46-50]^. Thereafter, RAF was identified as the upstream kinase of MEK in 1992 as well as the direct effector of RAS in 1993, which delineates the whole RAS/RAF/MEK/ERK signaling pathway^[51-55]^.

### The properties of components in the RAS/RAF/MEK/ERK signaling pathway

The first component of the RAS/RAF/MEK/ERK signaling pathway, RAS small GTPase has four isoforms HRAS, NRAS, KRAS4A, and KRAS4B, which are encoded by three genes^[2]^. RAS isoforms are highly homologous except their carboxyl-terminal hypervariable regions that undergo various post-translational modifications and anchor individual isoforms to different intracellular compartments^[56,57]^. Similar to other small GTPases, the activity of RAS proteins is primarily regulated by the GTP/GDP cycle. Active RAS proteins associate with GTP and adopt an active conformation that allows them to bind to and activate effectors, while inactive RAS proteins bound with GDP have altered conformation that impedes such interactions. In the GTP/GDP cycle of RAS proteins, the dislodging of GDP for GTP on RAS proteins is catalyzed by guanine exchange factor (GEF) proteins (i.e., Sos), whereas the hydrolysis of GTP into GDP by RAS proteins is facilitated by RAS GTPase activating proteins (GAPs) (i.e., NF1). Thus, the relative activity of GEF proteins *vs.* GAP proteins in cells shifts the GTP/GDP cycle and determines the output of RAS activity. However, it has to be noted that different RAS isoforms have differential propensities in activity regulation as well as tissue expression patterns and effector preferences^[58]^, which leads to their distinct physiological and pathological functions.

The direct effector of RAS, RAF/KSR family kinase includes three RAF isoforms (ARAF, BRAF, and CRAF) and two close pseudokinases (KSR1 and KSR2)^[59]^. All RAF proteins have highly homologous sequences and share three conserved regions (CR): CR1, CR2, and CR3. CR1 contains a RAS binding domain (RBD) and a Cys-rich domain^[60,61]^, while CR2 is characterized by Ser/Thr-rich sequence. CR3 is a putative kinase domain flanked with an N-terminal acidic motif (NTA) and a C-terminal regulatory tail^[62-64]^. In the inactive state, CR1 docks on CR3 and thus inhibits its kinase activity^[65,66]^. The association of RAS-GTP with CR1 relieves this inhibitory interaction and drives the dimerization of CR3, which further triggers the catalytic activity of CR3. Despite having a similar regulatory mechanism, RAF isoforms exhibit differential activities with an order as: BRAF > CRAF > ARAF. This likely arises from the diversity in their NTA motif and APE motif^[62,67]^. Different from RAF proteins, KSR1 and KSR2 have a coiled-coil fused sterile α-motif and Pro-rich stretch at their N-terminus, which are responsible for their plasma membrane recruitment, and lack the catalytic lysine in VAIK motif of kinase domain. By virtue of their low kinase activity and association with MEK and ERK, KSR proteins had been thought of as scaffolds for a long time^[68,69]^. However, recent studies have revealed that KSR proteins function as allosteric activators to stimulate the catalytic activity of RAF proteins through dimerization^[62,70,71]^. The allosteric activity of RAF/KSR family kinase is indispensable for their function^[72,73]^.

The second level of kinase in the RAS/RAF/MEK/ERK signaling pathway is MEK, a dual specific kinase, which includes two isoforms: MEK1 and MEK2^[74]^. These two kinases have a simple structure that consists of a short regulatory N-terminus and a canonical kinase domain. However, their regulatory mechanism(s) is not completely understood. At present, what we have learned about their regulation mainly includes: (1) the N-terminal regulatory region of MEK1/2 forms a helix that locks the kinase domain in an inactive conformation, and disrupting this inhibitory helix triggers the catalytic activity of MEK1/2^[75]^; (2) RAF/MEK and MEK/MEK interactions are required for MEK activation by RAF, in which MEK can be phosphorylated by RAF or itself^[67,76]^; and (3) even after activation by RAF, MEK needs to function as a dimer to phosphorylate downstream ERK^[76]^.

The terminal kinase of RAS/RAF/MEK/ERK signaling, ERK has two highly homologous isoforms (ERK1 and ERK2) as the upstream activator MEK does^[77]^. ERK isoforms comprise a central kinase domain flanked by short N- and C-terminal tails that regulate their activity, subcellular location, and substrate specificity^[78]^. ERKs also have redundant functions albeit with different expression patterns as MEKs do^[79]^. However, unlike RAFs and MEKs that have only certain substrates, ERKs recognize and phosphorylate over 200 proteins including transcription factors, protein kinases and phosphatases, and other structural or enzymatic proteins, which regulate diverse cell functions as well as compose a negative feedback loop for turning off the RAS/RAF/MEK/ERK signaling pathway^[80,81]^.

### The regulation of RAS/RAF/MEK/ERK signaling under physiological conditions

**Figure 1:**
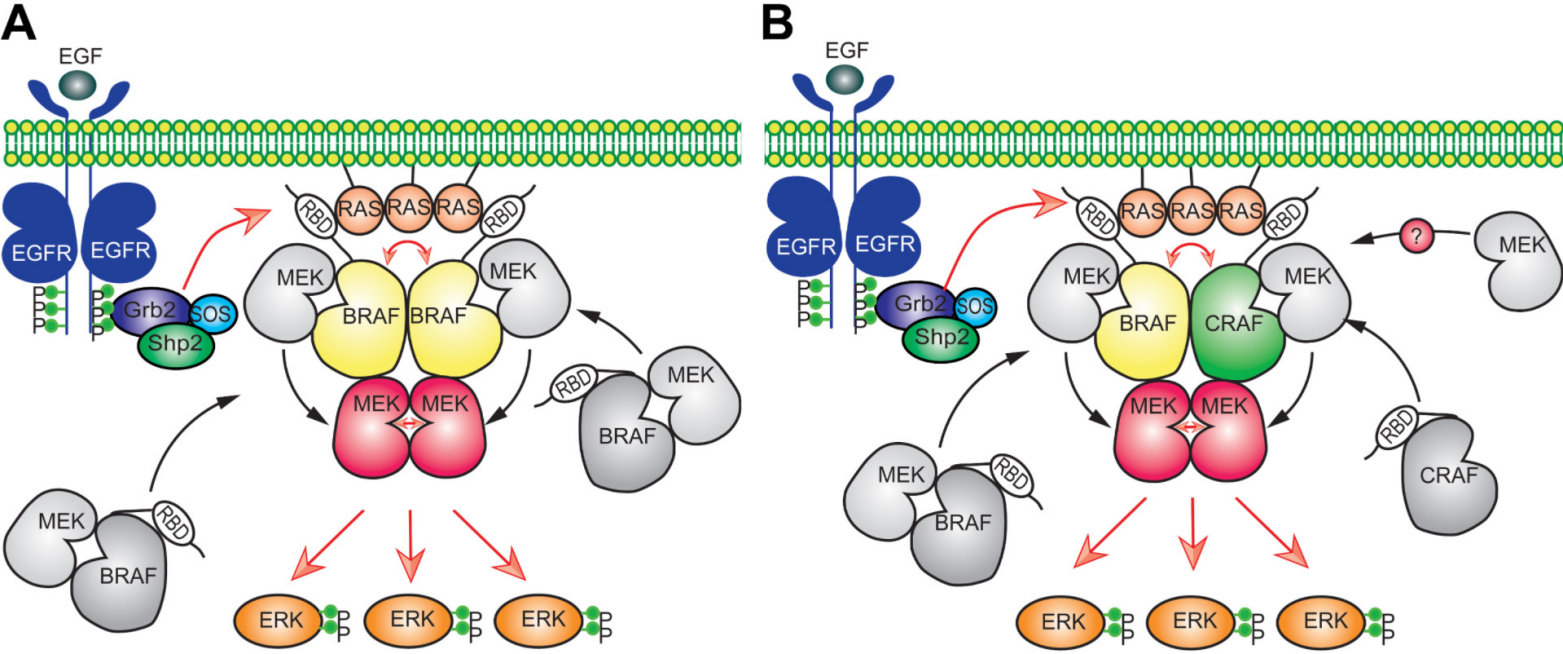
The regulatory mechanism of RAS/RAF/MEK/ERK signaling pathway. RAF and MEK exist as heterodimers in cytosol of quiescent cells (A). Upon Ras activation, these heterodimers are recruited to the plasma membrane where they form transient tetramers through side-to-side dimerization of RAF. Further, this side-to-side RAF dimerization activates RAF themselves and also loosens RAF/MEK heterodimers, which facilitate the MEK dimerization on the surface of RAF dimer. Subsequently, active RAF dimer phosphorylates MEK dimer docking on its surface or promotes intra-dimer MEK transphosphorylation. Thereafter, active MEK dimer docking on RAF dimer or released from RAF dimer phosphorylates and activates ERK. Since, unlike BRAF, CRAF and ARAF do not heterodimerize with MEK in inactive state, how MEK is delivered to CRAF and ARAF for activation remains unknown in current studies (B). EGF: Epidermal growth factor; RBD: RAS binding domain.

The regulation of RAS/RAF/MEK/ERK signaling is very complex and involves complicated intermolecular interactions and phosphorylation events^[20,82,83]^. Although the framework of RAS/RAF/MEK/ERK signaling was discovered almost three decades ago, the approximate regulatory network for this signaling was uncovered only recently [Figure 1]. In quiescent cells, RAF proteins are immobilized in inactive conformation by 14-3-3 dimers through intramolecular association and form face-to-face heterodimers with MEKs^[84,85]^. The engagement of RTKs with their ligands triggers their kinase activity and results in the autophosphorylation of intracellular carboxyl-tails, which serve as docking sites for recruiting Grb2 and Sos1 to plasma membrane^[86]^. Further, active Sos1 proteins on the plasma membrane exert their GEF activity towards RAS, which in turn actives RAS proteins to recruit RAF/MEK heterodimers to the plasma membrane through the RBDs of RAF proteins^[87-89]^. Since active RAS proteins form oligomers or nanoclusters, RAF proteins are enriched on the plasma membrane, which promotes their side-to-side dimerization^[90-93]^. RAF dimerization on the plasma membrane can be further enhanced by the switching of intramolecular into intermolecular associations of 14-3-3 dimers with RAF proteins, which eventually turns on the catalytic activity of RAF proteins^[64,94]^. Active RAF dimers autophosphorylate their activation loops and, at the same time, loosen RAF/MEK interaction to facilitate the assembly of MEK homodimers on their surface^[62,76]^. Once MEK proteins form face-to-face homodimers that dock on RAF dimers, RAF proteins will phosphorylate their activation loops or trigger the cross-phosphorylation of two protomers in MEK homodimers, which ultimately activates MEK dimers^[76]^. The assembly of MEK dimers on the surface of RAF dimers also reversely stabilizes RAF dimers, which illustrates why active MEKs can transactivate RAFs^[62,95]^. Subsequently, active MEK dimers release from or continuously dock on RAF dimers to phosphorylate ERKs^[76]^. Thereafter, phospho-ERKs travel to different intracellular compartments and phosphorylate their substrates as monomers or dimers, which gives rise to diverse cell responses^[3,76]^. Under physiological condition, active ERKs also drive a negative feedback loop through inhibitory phosphorylating EGFR, Sos1, and RAF, as well as upregulating dual-specificity phosphatases and Sprouty proteins, which shuts off the whole signaling pathway and returns cells back to quiescent status^[80,81,96]^. It must be noted that this general regulatory network may be fine-tuned by the properties of components that constitute this signaling pathway. For instance, in contrast to BRAF, CRAF and ARAF do not form heterodimers with MEK in the inactive state^[84]^, and how MEKs are delivered to these two RAF proteins for activation remains an intriguing question in current studies.

## HYPERACTIVE RAS/RAF/MEK/ERK SIGNALING IN CANCERS

**Figure 2:**
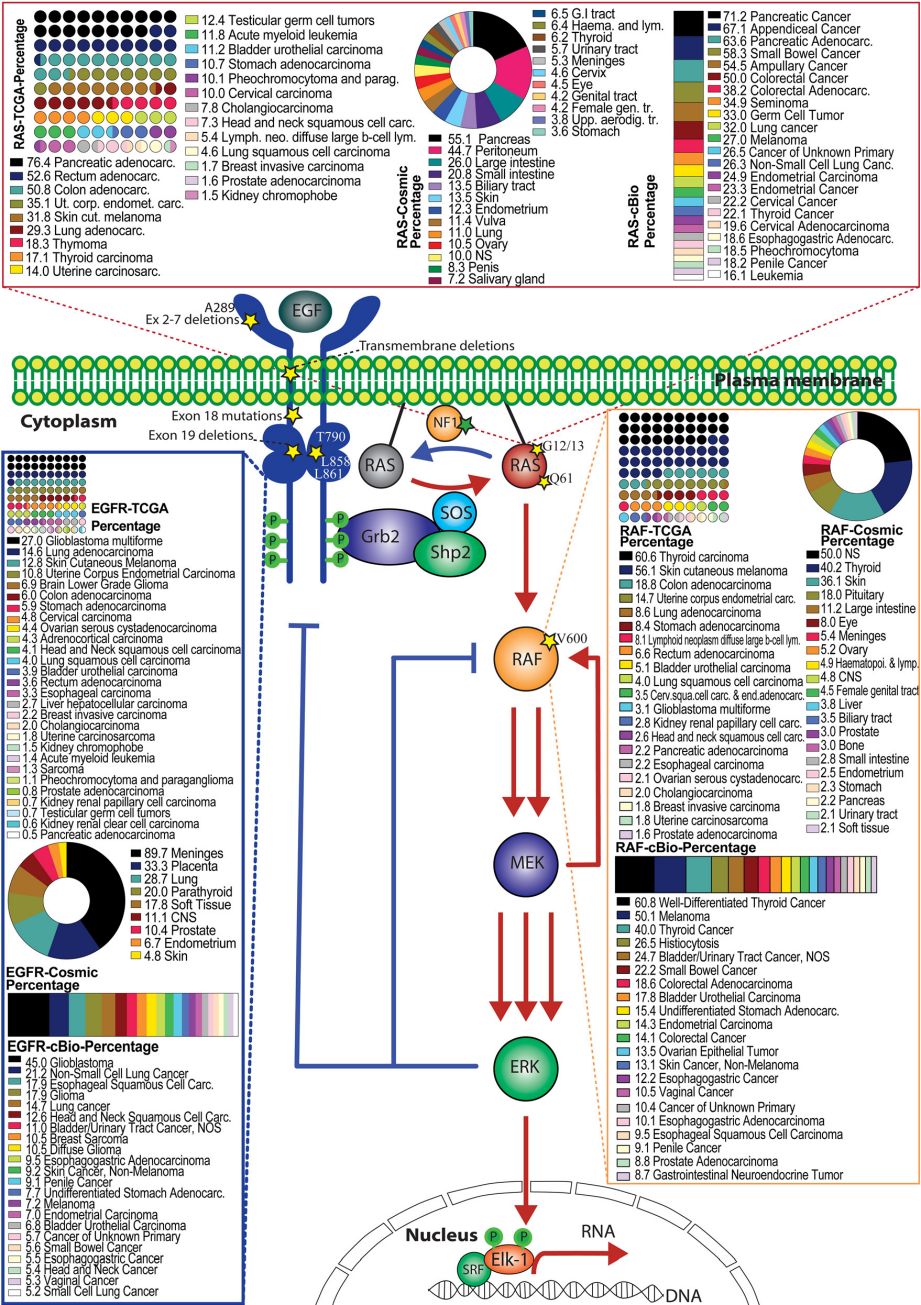
Genetic mutations that hyperactivate the RAS/RAF/MEK/ERK signaling pathway in human cancers. The RAS/RAF/MEK/ERK signaling is initiated on the plasma membrane where engagement of EGFR by its ligand activates Sos. In turn, Sos functions as a RAS-GEF to catalyze the GTP loading on RAS. Subsequently, RAS-GTP triggers the RAF/MEK/ERK kinase cascade and ultimately activates ERK. As the terminal kinase of this signaling cascade, active ERK phosphorylates numerous substrates and induces diverse cell responses. This signaling cascade is tightly regulated under physiological conditions through complicated mechanisms that include MEK-mediated positive feedback (red arrows) as well as NF1-driven GTP hydrolysis and ERK-mediated negative feedback (blue arrows). In cancer cells, this signaling cascade is mainly hyperactivated by genetic mutations on EGFR, NF1, RAS, and RAF. The hotspots for oncogenic mutations in these genes are labeled as yellow stars. The mutational prevalence of these genes in different type of cancers (calculated from TCGA, cBio, and COSMIC databases) are shown in boxes. TCGA data are shown in small circles forming square, while COSMIC data and cBio data are shown as a donut plot and a box graph, respectively. Numbers on the legends indicate the percentages of mutations in the particular cancer types. EGF: Epidermal growth factor.

The pivotal role of RAS/RAF/MEK/ERK signaling in cell biology has been highlighted by studies in developmental disorders and cancers even before the delineation of the entire pathway. Aberrant activation of RAS/RAF/MEK/ERK signaling by germline mutations causes developmental disorders termed as RASopathies that include neurofibromatosis type 1 (NF1), Noonan syndrome, Castello syndrome, and cardio-facio-cutaneous syndrome^[4]^. In contrast, dysregulating this pathway by somatic mutations results in cancers^[19,20,97]^. In this review, we focus on somatic mutations that directly hyperactivate RAS/RAF/MEK/ERK signaling in cancers [Figure 2].

### RTK alterations

The first prominent target of oncogenic alterations that aberrantly activate RAS/RAF/MEK/ERK signaling is RTKs, particularly EGFRs. It is well established that EGFRs are activated through ligand engagement-driven dimerization of intracellular kinase domain^[98-100]^. Genetic alterations that trigger or bypass this process would hyperactivate EGFRs and thus drive tumorigenesis^[101]^. According to underlying activating mechanisms, oncogenic EGFR alterations can be classified as three groups: (1) overexpression of EGFRs that drives EGFR oligomerization independent of ligand association, which occurs in non-small cell lung cancer, glioblastoma, breast cancer, and pancreatic cancer^[101-103]^; (2) mutations in the extracellular ligand-binding domain (ECD), transmembrane domain (TMD), or juxtamembrane domain (JMD) of EGFRs that enhance the ligand affinity or bypass ligand binding, which are resident in gliomas, lung adenocarcinoma, breast cancer, and colorectal cancer^[102,104]^; and (3) constitutively active mutations in the kinase domain of EGFRs that mainly comprise β3-αC loop deletions (exon19del) and activation loop mutations (L858 in exon 21), which represent the majority of EGFR mutations in lung cancer and glioblastoma^[105,106]^. Targeting oncogenic EGFR alterations with neutralizing antibodies and TKIs has significantly changed the landscape of cancer treatment in recent years and has been comprehensively reviewed by many groups^[107-109]^.

### NF1 mutations

The second noticeable gene whose mutations hyperactivate RAS/RAF/MEK/ERK signaling in cancer is *NF1* that encodes a RAS-GAP and functions as a tumor suppressor. NF1 was originally discovered in neurofibromatosis type 1, a common developmental disorder caused by germline mutations of *NF1* gene. Somatic mutations of NF1 are responsible for ~10% of melanoma, lung cancer, glioblastoma, and bladder cancer cases^[10]^. Interestingly, NF1-mutated cancers have a heavier mutational burden and thus exhibit a better response to immunotherapy than those with RAS or RAF mutations^[110,111]^.

### RAS mutations

*RAS* is the most frequently mutated oncogene and has an impact on over 30% of cancers^[112]^. However, RAS mutations have isoform and tissue preferences, resulting in the enrichment of specific RAS mutations in certain cancers. For example, KRAS mutations are highly prevalent in pancreatic cancers (~90%), colorectal cancers (~35%), and lung cancers (~20%), while NRAS mutations exist in melanoma (~20%) and thyroid cancers (~20%)^[113,114]^. Furthermore, RAS mutations have two structural hotspots on G12/13 and Q61, which impairs RAS-GAP association or inhibits intrinsic GTPase activity of RAS proteins, and thus extends the half-life of RAS-GTP. Due to the extremely high affinity with GTP and the lack of a proper binding pocket for docking small molecular inhibitors, most RAS mutants are not druggable except G12C mutants that could be targeted with covalent small molecular inhibitors through docking in the switch II pocket and cross-linking with Cys12^[115-119]^. Therefore, targeting the downstream RAF/MEK/ERK kinase cascade has become a primary approach for treating cancers with non-G12C RAS mutations.

### RAF mutations

Although the N-terminal truncated RAF (CRAF or RAF1) was first discovered as a retroviral oncogene in the early 1980s, oncogenic RAF mutations had not been observed in cancers until 2002^[120]^. Moreover, it is BRAF and not CRAF, ARAF, or KSR that is frequently mutated in cancer genomes^[121]^. A single point mutation (V600E) in the activation loop of BRAF represents > 90% of all cancer-related RAF mutations^[17,20]^. Mechanistic studies have suggested that oncogenic RAF mutations might be classified as three groups: (1) highly constitutively active mutants such as BRAF(V600E) that directly phosphorylate MEK and thus activate downstream signaling cascade; (2) kinase-dead or -impaired mutants such as BRAF(V471F) that function as allosteric activators and switch on downstream signaling through triggering the catalytic activity of wild-type RAFs; and (3) intermediate active mutants such as BRAF(G469A) that have enhanced dimer affinity and directly turn on downstream signaling as homodimers^[122-124]^. However, there are some unique RAF mutants such as BRAF(ΔNVTAP), BRAF(L505H), and BRAF(LLR^ins506^) that do not fall into any of these categories^[67,125-129]^, suggesting that oncogenic RAF mutations have diverse characters and complicated functional modes. Similar to RAS mutations, RAF mutations also have cancer-type preferences. Although RAF mutations exist in only ~7% of all cancers, they are highly prevalent in melanoma (~50%), thyroid cancer (~45%), and Langerhans cell histiocytosis (~50%)^[17,20,130]^.

In contrast to dominant mutations of EGFR, NF1, RAS, and RAF, there are some rare activating mutations of Sos, MEK, and ERK in cancer genomes that constitutively turn on RAS/RAF/MEK/ERK signaling. The functional analysis of these rare mutations in cancer biology has been reviewed before^[20,131]^, and we do not discuss them again here.

## TARGET HYPERACTIVE RAS/RAF/MEK/ERK SIGNALING IN CANCERS WITH RAF INHIBITORS

Since oncogenic alterations that aberrantly activate RAS/RAF/MEK/ERK signaling mainly occur on RAFs, particularly BRAF or upstream, this family of kinases has been considered an ideal target for cancer drug development. Given the high prevalence of BRAF(V600E) in cancer genomes, Tsai *et al.*^[21]^ resolved its high-resolution crystal structure and further utilized a structure-guided drug discovery approach to identify a potent and selective small molecule inhibitor of BRAF(V600E), PLX4720. This inhibitor exhibited a strong antitumor activity on BRAF(V600E)-harboring melanoma *in vitro* and *in vivo*^[21]^, and its analog with more favorable pharmacokinetics, vemurafenib (PLX4032), was first approved for treating advance melanomas and then for other cancers with BRAF(V600E) mutation [Table 1]^[24,132,133]^. In clinical therapy, vemurafenib exhibited an exceptional efficacy with manageable side effects on cancers with BRAF(V600E) mutation as a single agent or combined with cobimetinib (MEK inhibitor), although drug resistance occurs after 6-8 months of administration.

**Table 1:** Small molecule RAF inhibitors that have been applied to clinical cancer therapy or are undergoing development

**Drug name/aliases**		**Structure**	**Remark**
First generation (Type I1/2) RAF inhibitor	Vemurafenib PLX4032 Zelboraf	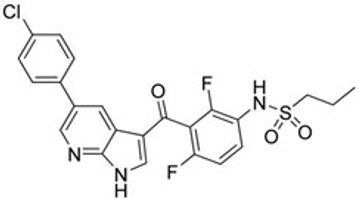	**Classification: **Type I1/2 RAF inhibitor. **PFS: **6.8 months (single agent), 9.9 months (combined with cobimetinib). **ORR: **45% (single agent), 68% (combined with cobimetinib). **OS: **15.9 months (median, single agent), 81% at 9 months (combined with cobimetinib). **Side effects: **new primary cutaneous malignancies (lower secondary cutaneous cancers in combination with MEKi). Clinically available (FDA approved in 2011 for single agent and in 2015 for combination with cobimetinib)
	Dabrafenib GSK-2118436 TAFINLAR	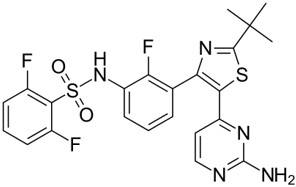	**Classification: **Type I1/2 RAF inhibitor. **PFS: **8.8 months (single agent), 11.0 months (combined with trametinib). **ORR:** 53% (single agent), 69% (combined with trametinib). **OS (median): **18.7 months (single agent), 25.1 months (combined with trametinib). **Side effects: **new cutaneous and non-cutaneous malignancies (lower secondary cutaneous cancers in combination with MEKi). Clinically available (FDA approved in 2014 for single agent and in 2018 for combination with trametinib) (refer to trametinib)
	Encorafenib Lgx818 Braftovi	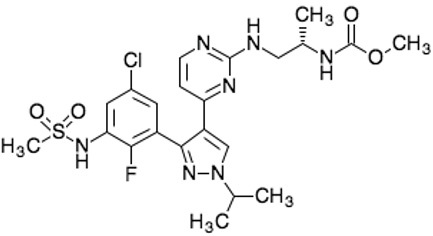	**Classification: **Type I RAF inhibitor. **PFS: **9.6 months (single agent), 14.9 months (combined with binimetinib). **ORR: **52% (single agent) (better than vemurafenib within the same cohort 41%), 64% (combined with binimetinib). **OS (median): **23.5 months (single agent), 33.6 months (combined with binimetinib). **Side effects: **new primary malignancies, cutaneous and non-cutaneous. Clinically available (FDA approved in 2018 for combination with binimetinib and in 2020 for combination with cetuximab)
Second generation (Type II) RAF Inhibitor	PLX8394	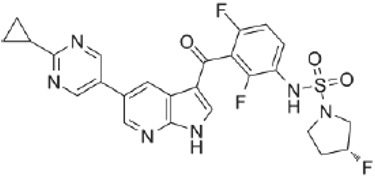	**Classification:** RAF dimer breaker. Induces a conformation unfavorable for dimerization. **Partial response rate: **22%.** Side effects: **diarrhea. Phase I/II is recruiting for advanced unresectable solid tumors (NCT02428712)
	CCT3833/BAL3833	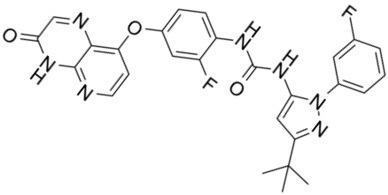	**Classification:** Pan-RAF/SRC kinase inhibitor. Phase I for solid tumors was completed (NCT02437227)
	LY3009120	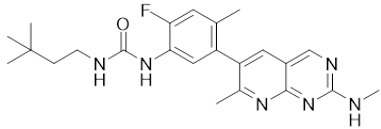	**Classification: **Pan-RAF, RAF dimer inhibitor. Inhibits both RAF protomers. **Side effects: **fatigue, nausea, low appetite, and rash. Phase I for advanced or metastasized tumors was terminated (NCT02014116)
	BGB-283 Lifirafenib BEIGENE-283	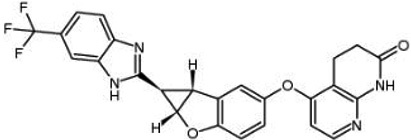	**Classification:** Pan-RAF inhibitor. **Side effects: **hypertension and fatigue. Phase I is recruiting for advanced or refractory solid tumors (NCT03905148)
	Belvarafenib	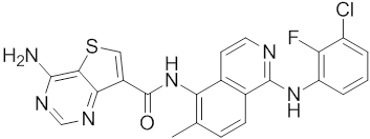	**Classification:** Pan-RAF inhibitor. **Side effects: **rash, dermatitis, acneiform, and pyrexia. Phase I is recruiting for NRAS mutant advanced melanoma (NCT04835805)
	TAK-580	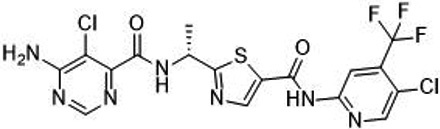	**Classification: **Pan-RAF inhibitor.** Side effects: **rash and increased bilirubin in once-a-week treatment for ST or melanoma detected in Phase I. Phase I is recruiting for gliomas and other tumors (NCT03429803)
Advanced RAF inhibitor	Trametinib Gsk1120212	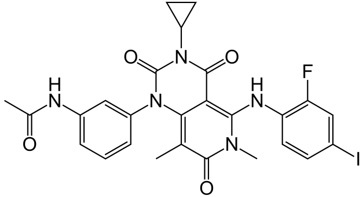	**Classification:** RAF/MEK heterodimer breaker. Binds to the RAF/MEK interface. Prevents RAF/MEK association and MEK phosphorylation. **PFS: **4.8 months (single agent), 11.0 months (combined with dabrafenib)** ORR: **22% (single agent), 68% (combined with dabrafenib). **OS (median): **15.6 months (single agent), 25.1 months (combined with dabrafenib). **Side effects: **hemorrhage, colitis, and venous thromboembolism. Clinically available (FDA approved in 2013 for single agent, also approved for combination therapy with dabrafenib in 2018)
	RO5126766	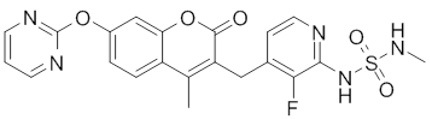	**Classification:** RAF/MEK complex inhibitor. Docks on RAF/MEK dimer interface and traps RAF/MEK complex in inactive state. **Side effects: **rash, elevated CPK, and diarrhea. Phase I is recruiting for solid tumors and multiple myeloma (NCT02407509)
	Trametiglue	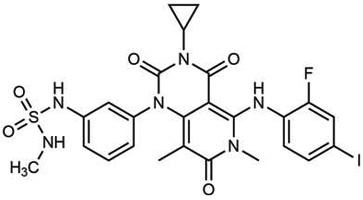	**Classification:** RAF/MEK complex inhibitor. Derived from structure of trametinib. Docks on RAF/MEK dimer interface and traps RAF/MEK complex in inactive state. Clinical trial has not begun

Three first-generation RAF inhibitors have been developed and applied to treat BRAF(V600E)-harboring cancers as monotherapies or combinatory therapies with MEK inhibitors. The major problem with these inhibitors is their paradoxical effect in which RAF inhibitors bind to one protomer and transactivate the other protomer in RAF dimers. To resolve this issue, the second-generation RAF inhibitors that include RAF dimer breakers and pan-RAF inhibitors have been developed and are undergoing clinical evaluations. However, all these RAF inhibitors belong to ATP competitive kinase inhibitors (Type I or II inhibitors). Some advance RAF inhibitors that target intermolecular interactions in RAF/MEK complex are now under development, which may have better efficacies and less side effects in targeted cancer therapy.

Shortly after the discovery of vemurafenib, a second RAF inhibitor, dabrafenib (GSK2118436), was developed by the GSK group [Table 1]^[22]^. Although dabrafenib is a reversible competitive inhibitor similar to vemurafenib, it is more selective for BRAF(V600E) over wild-type BRAF and other RAF kinases than vemurafenib^[22]^. Moreover, dabrafenib has a similar potency against BRAF(V600E) and BRAF(V600K)^[134]^. In clinical trials, dabrafenib exhibited a similar efficacy as well as side effects for treating advance melanoma with BRAF(V600E) mutation, but better activity on brain metastasis in contrast to vemurafenib^[135-137]^, although no direct comparison between these two inhibitors has been carried out to reach a definitive conclusion. As for vemurafenib, the efficacy of dabrafenib was further extended when combined with a MEK inhibitor (trametinib)^[138,139]^, which led to approval of dabrafenib plus trametinib, as a combinatorial agent for treating advance melanoma harboring BRAF(V600E/K) mutations in clinical therapy.

Although the combinatorial regimens of vemurafenib plus cobimetinib and dabrafenib plus trametinib have achieved promising outcomes at the initial therapeutic phase, drug resistance and adverse effects limit their effectiveness. To achieve better efficacy and tolerability of RAF inhibitors through optimizing their pharmacological properties, a third RAF inhibitor, encorafenib (LGX818), was developed by the Novartis group [Table 1]^[23]^. In contrast to vemurafenib and dabrafenib, this inhibitor has over 10-fold slower off-rate and a much shorter serum half-life^[23,140]^, which delays drug resistance and improves tolerability. Furthermore, encorafenib has much higher specificity than vemurafenib and dabrafenib, and it targets not only V600E but also V600K and V600D^[141]^. In clinical trials, encorafenib achieved significantly longer progression-free survival and fewer adverse effects on advance melanoma with V600 mutations as both monotherapy and combination therapy with the MEK inhibitor binimetinib, compared with vemurafenib^[26,27,142]^. In addition, encorafenib exhibited an excellent efficacy against metastatic colorectal cancers with BRAF(V600E) mutation when combined with cetuximab^[143]^. These trials led to the approval of encorafenib plus binimetinib or cetuximab for treating advance BRAF(V600E)-harboring cancers. Therefore, vemurafenib, dabrafenib, and encorafenib constitute the first-line agents against advance cancers with BRAF(V600E) mutation in current clinical therapy, which significantly changes the landscape of cancer therapy.

## DRUG RESISTANCE IN TARGETED THERAPIES WITH RAF INHIBITORS

Although the first-generation RAF inhibitors achieved promising outcomes on treating BRAF(V600E)-harboring cancers, their efficacy is abrogated by quick-rising resistance within a median time of 6-7 months^[24-27]^. To understand molecular mechanisms that underlie RAF inhibitor resistance, extensive studies have been carried out. The overexpression/upregulation of RTKs such as PDGFRβ and EGRF was reported as the first type of alterations that led to RAF inhibitor resistance^[144-146]^, which activates RAS and turns on CRAF-MEK-ERK signaling, and hence bypasses BRAF(V600E)-dependence for cancer cell growth. Downstream of RTKs, RAS mutations were identified as the second cause for RAF inhibitor resistance^[144,147]^, which function similarly as RTK alterations. In other words, once cancer cells possess a high level of active Ras, the drug-loaded BRAF(V600E) will dimerize with CRAF and trigger its catalytic activity^[28-30,32,33]^, which has been termed as the paradoxical effect of RAF inhibitors. Furthermore, N-terminal alternative splicing and overexpression of BRAF(V600E) were discovered in drug-resistant cancer patient samples^[31,148,149]^. This type of alterations enhances BRAF(V600E) homodimerization and impairs drug engagement. Unlike drug resistance that occurs in EGFR mutation-driven cancers or BCR-Abl-induced leukemia, secondary mutations such as gate-keeper mutations that cause drug resistance are rare in BRAF(V600E)-driven cancers. Thus far, only two cases (L505H and L514V) have been reported to confer drug resistance as secondary mutations in BRAF(V600E)-driven cancers^[127,150]^. As the direct effectors of RAF proteins, MEK also contributed to RAF inhibitor resistance, whose constitutively active mutations were identified in 3%-5% of cases^[151,152]^. Altogether, ~80% of drug resistant cases in BRAF(V600E)-harboring cancers arise from pathway reactivation of ERK signaling^[151,152]^. Although several mechanisms other than ERK reactivation have been suggested to be responsible for ~20% of drug resistance cases^[153-156]^, none of them contributes to a significant portion of those cases.

Given the dominant role of RAF dimerization in the pathway reactivation of cancer cells upon RAF inhibitor treatment, a “monomer-dimer” hypothesis has been suggested to explain the majority of drug resistance cases in targeted therapy^[31,157]^. In this hypothesis, BRAF(V600E) was thought as a monomer to phosphorylate MEK, which has a high affinity for RAF inhibitors and hence can be inhibited by these drugs. In contrast, once BRAF(V600E) forms homodimers or heterodimers with other RAF proteins, its affinity for RAF inhibitors dramatically decreases and hence its catalytic activity cannot be blocked effectively by these drugs. This hypothesis was further strengthened by findings that BRAF mutants with in-frame deletions in β3-αC loop have elevated dimer affinity and are resistant to RAF inhibitors^[126]^. However, it was also challenged by some emerging findings, which have indicated that BRAF(V600E) exists as dimers or oligomers in cells even though its dimer affinity is not as high as that of BRAF(V600E) variants with a truncated N-terminus or BRAF mutants with an in-frame deletion of β3-αC loop, and the monomeric BRAF(V600E) variant has no catalytic activity *in vitro* and *in vivo*^[67,158-160]^. Therefore, how elevated dimer affinity of BRAF confers RAF inhibitor resistance and the root of RAF inhibitor resistance are not defined yet.

Recently, we and other groups identified a novel group of oncogenic BRAF mutants with in-frame insertions of LLR or VLR in the αC-β4 loop^[129,161,162]^. We found that these unique BRAF mutants had much lower dimer affinity than their wild-type counterpart or BRAF(V600E). Similar to other constitutively active BRAF mutants with low dimer affinity^[67,163,164]^, they lost their catalytic activity upon purification from cells, which can be rescued by GST-fusion-enhanced dimerization. However, they exhibited a very strong resistance to all RAF inhibitors in clinical therapy or undergoing clinical trials *in vitro* and *in vivo*, indicating that the dimer affinity of RAF proteins is not directly correlated to RAF inhibitor resistance, although an enhanced dimerization could cause drug resistance. To uncover the molecular basis responsible for RAF inhibitor resistance, we determined the conformational alterations in these BRAF mutants through structure modeling and molecular dynamic simulation and found that the inserted LLR or VLR residues facilitated an assembly of large hydrophobic network that includes and stabilizes the regulatory spine (R-spine), a typical hydrophobic architecture of active protein kinases^[165,166]^. Since RAF inhibitor engagement broke the R-spine of BRAF^[21,167,168]^, a stabilized R-spine would remarkably impair drug loading. Indeed, these BRAF mutants have much less affinity for RAF inhibitors, suggesting instead that a stabilized R-spine leads to drug resistance. Given that R-spine can be stabilized by different mutations via various manners, we then determined whether other BRAF mutants with a stabilized R-spine such as L485F and L505H^[127,128]^ were resistant to RAF inhibitors, and found that all these mutants were resistant to RAF inhibitors with different extents related to their R-spine stability. The R-spine stability can also explain why BRAF(V600E) splicing variants or other mutants with high dimer affinity are resistant to RAF inhibitors: a strong dimerization would lead to a cooperative assembly of R-spine in two protomers of a dimer, and hence R-spine held in a tight dimer is more stable than that in a lax dimer, which impairs drug engagement. Altogether, our findings indicate that, instead of dimer affinity, R-spine stability is the root of RAF inhibitor resistance of RAF proteins.

## DEVELOPING NEXT-GENERATION RAF INHIBITORS FOR OVERCOMING DRUG RESISTANCE

Since the majority of drug resistance in targeted therapies with first-generation RAF inhibitors was caused mainly by enhanced homo/heterodimerization of RAF proteins, two types of second-generation RAF inhibitors have been developed to resolve these issues: RAF dimer breakers (i.e., PLX8394) and pan-RAF inhibitors (i.e., BAL3833, LY3009120, BGB283, belvarafenib, and TAK-580) [Table 1]. RAF dimer breakers induce a conformation unfavorable for dimerization upon engagement with BRAF(V600E)^[169]^ and hence impair homodimerization driven by N-terminal deletion or overexpression of BRAF(V600E). This type of inhibitors has exhibited promising efficacy *in vivo* against tumors that have alternative splicings or upregulation of BRAF(V600E) or other BRAF mutants with moderate dimer affinity [i.e., BRAF(G469A) and BRAF fusion mutants] and thus are resistant to first-generation RAF inhibitors^[170,171]^. Currently, this type of inhibitors is undergoing evaluation in stage I/II clinical trials. Unlike RAF dimer breakers, pan-RAF inhibitors are able to bind and block the catalytic activity of both protomers in RAF dimers^[168,172,173]^. This type of inhibitors may replace the Phe residue from DFG motif to form a much more stable hydrophobic network in RAF protomers upon engagement and is able to block the activity of RAF homo/heterodimers even with very high dimer affinity such as in BRAF(ΔNVTAP)^[126]^. Consistently, pan-RAF inhibitors have exhibited good efficacy against tumors that have active RAS mutations or high-affinity RAF dimer mutants in animal models, particularly when combined with MEK or ERK inhibitors^[174-176]^. However, since pan-RAF inhibitors target both wild-type and mutant RAF proteins, their therapeutic index should not be high as a single agent.

Although the second-generation RAF inhibitors might overcome resistance arising from RAF dimerization enhanced by BRAF alterations or active RAS, they can hardly block the activity of some emerging oncogenic BRAF mutants such as L505H and LLR^ins506^^[129]^. As discussed above, both first- and second-generation RAF inhibitors disassemble the R-spine upon engagement and are unable to associate with RAF mutants possessing highly stabilized R-spines. Therefore, there is an unmet need for developing more effective RAF inhibitors that overcome drug resistance caused by these novel RAF alterations. Since the first two generations of RAF inhibitors belong to type I_1/2 _or II kinase inhibitors, the development of next-generation RAF inhibitors has been switched to type III and IV inhibitors that target the catalytic cleft or allosteric sites of RAF kinase domain, and thus may have much higher specificity and fewer off-target effects [Table 1]. In particular, given the unique activating and catalytic mechanisms of RAF family kinases, targeting RAF/RAF and RAF/MEK interactions has become an attractive strategy for designing novel RAF inhibitors^[76]^. Although no small molecular inhibitors that target RAF/RAF dimer interface have been developed at present, several groups have demonstrated that disrupting RAF/RAF dimers by using peptide inhibitors can effectively block hyperactive ERK signaling and thereby inhibit the growth of cancer cells harboring active RAF or RAS mutations^[177-179]^, indicating that the RAF/RAF dimer interface is indeed a valid target for drug development. As for RAF/MEK interaction, it is dynamically altered in the process of MEK phosphorylation by RAF, as described above. Therefore, breaking the RAF/MEK complex or trapping the RAF/MEK complex in an inactive state would inhibit the catalytic activity of RAF. The first FDA-approved MEK inhibitor for cancer therapy, trametinib has been shown as a RAF/MEK heterodimer breaker, which binds to the interface of MEK and impairs RAF/MEK association as well as subsequent phosphorylation of MEK by RAF^[76,180,181]^. On the other hand, RO5126766, a RAF/MEK dual inhibitor that docks onto the RAF/MEK interface but prevents conformational alteration of the RAF/MEK complex, also blocks the MEK phosphorylation by RAF as trametinib does^[182]^. This allosteric inhibitor has exhibited promising activity against various cancers harboring RAS mutations in Phase I clinical trials^[183-185]^. Using a similar strategy, Khan *et al.*^[181]^ developed a new RAF/MEK dual inhibitor, trametiglue, by modifying trametinib, which still docks onto the RAF/MEK interface and traps the RAF/MEK complex in an inactive state. In contrast to RO5126766, trametiglue has orders of magnitude higher potency and much slower off-rate kinetics, which results in better long-term inhibitory activity on both BRAF- and RAS-mutated tumors, although its clinical efficacy needs further evaluation. Altogether, these allosteric RAF inhibitors that target RAF/RAF or RAF/MEK interfaces without altering R-spine upon engagement could have higher efficacies against cancers harboring R-spine-stabilized RAF mutants, although they have not been tested on such cancers yet.

Besides these validated allosteric sites involved in RAF/RAF and RAF/MEK interactions, whether there are other allosteric cavities suitable for small molecule docking and hence developing advance RAF inhibitors remains unknown. Nevertheless, the high-resolution structures of RAF proteins and RAF/MEK complexes resolved in recent years^[21,29,64,84,85,94,186]^ make possible *in silico* investigation of such allosteric sites and will accelerate the development of RAF inhibitors, particularly isoform-specific inhibitors through computation-aided molecular docking and optimization.

## CONCLUSION

Targeting hyperactive RAS/RAF/MEK/ERK signaling with RAF inhibitors has achieved promising outcomes for treating cancers harboring BRAF(V600E) mutation. However, both intrinsic and acquired resistance have severely restricted their applications in cancer therapy. With a deeper understanding of regulatory mechanism(s) of RAF family kinases, next-generation RAF inhibitors that target allosteric sites on kinases and thus have longer efficacy, broader coverage, and fewer off-target effects are being developed, which sheds a light on overcoming emerging drug resistance in current cancer therapy.

## DECLARATIONS

### Copyright

© The Author(s) 2021.
